# Challenges in conducting community-driven research created by differing ways of talking and thinking about science: a researcher's perspective

**DOI:** 10.3402/ijch.v72i0.21232

**Published:** 2013-08-05

**Authors:** Amy Colquhoun, Janis Geary, Karen J. Goodman

**Affiliations:** Department of Public Health Sciences, School of Public Health, University of Alberta, Edmonton, Alberta, Canada; Centre of Excellence in Gastrointestinal Inflammation and Immunity Research, Department of Medicine, University of Alberta, Edmonton, Alberta, Canada

**Keywords:** Aboriginal health, *Helicobacter pylori*, cancer, circumpolar regions

## Abstract

Increasingly, health scientists are becoming aware that research collaborations that include community partnerships can be an effective way to broaden the scope and enhance the impact of research aimed at improving public health. Such collaborations extend the reach of academic scientists by integrating a variety of perspectives and thus strengthening the applicability of the research. Communication challenges can arise, however, when attempting to address specific research questions in these collaborations. In particular, inconsistencies can exist between scientists and community members in the use and interpretation of words and other language features, particularly when conducting research with a biomedical component. Additional challenges arise from differing perceptions of the investigative process. There may be divergent perceptions about how research questions should and can be answered, and in expectations about requirements of research institutions and research timelines. From these differences, misunderstandings can occur about how the results will ultimately impact the community. These communication issues are particularly challenging when scientists and community members are from different ethnic and linguistic backgrounds that may widen the gap between ways of talking and thinking about science, further complicating the interactions and exchanges that are essential for effective joint research efforts. Community-driven research that aims to describe the burden of disease associated with *Helicobacter pylori* infection is currently underway in northern Aboriginal communities located in the Yukon and Northwest Territories, Canada, with the goal of identifying effective public health strategies for reducing health risks from this infection. This research links community representatives, faculty from various disciplines at the University of Alberta, as well as territorial health care practitioners and officials. This highly collaborative work will be used to illustrate, from a researcher's perspective, some of the challenges of conducting public health research in teams comprising members with varying backgrounds. The consequences of these challenges will be outlined, and potential solutions will be offered.

Scientists attempt to gain new knowledge by applying their expertise to learn more about the world around them. Historically, with the exception of necessary practical requirements such as funding applications and access to data, this work was typically done in isolation: scientists from universities conducted research and worked to publish their results in scientific journals. This process required little interaction between the scientists producing research results and the community members or stakeholders who made use of this information. In recent years, it has become widely recognized that there are limits to the utility of research conducted in isolation. Instead, it is preferable to build “context-sensitive” knowledge through the pursuit of research that will have a real-world impact; this process will help to grow knowledge in a meaningful way (1) and is particularly relevant for public health research.

One way that scientists build context-sensitive knowledge is by developing research collaborations that include community partnerships. Such collaborations have been shown to produce a variety of benefits (2–4). These benefits include access to communities, individuals or datasets that may be optimal for addressing specific research questions. Researcher–community partnerships are also a means to create other benefits such as building capacity, generation of new research questions, and broadening understanding and knowledge amongst all partners: community members are able to learn about the scientific process and researchers are able to gain insights into community perspectives and other forms of knowledge creation (2).

Community–university collaborations have become common in the field of public health and have been found to be an effective way to broaden the scope and enhance the impact of public health research. Such collaborations extend the reach of academic scientists by integrating a variety of perspectives and thus strengthening the applicability of the research (1–3). Comprehensive collaborations between researchers and community members throughout the design and conduct of research can work to “enrich knowledge, address and help solve critical societal issues, and contribute to the public good” (2). Ensuring that community members and end-users are participants in the research process from the beginning increases the chance that research results will be meaningful and useful for all stakeholders.

Despite the benefits to participating in collaborative research, there are some challenges. Communication challenges can arise when attempting to address specific research questions in these collaborations. This article describes some of the challenges that occur when there are inconsistencies in the use of language and definitions, and when scientists and community members have differing perceptions of the investigative process, particularly when the research includes biomedical frameworks. To illustrate these issues, we present a case study highlighting a collaborative public health research program conducted in northern Canada to address community concerns related to health risks from chronic *Helicobacter pylori* infection and, in particular, its most serious disease consequence—stomach cancer. Finally, we discuss potential consequences of these communication challenges and suggest potential solutions that may reduce future challenges when working in collaborative researcher–community settings. In highlighting distinctions between researchers and community members in what follows, we are not implying that either group lacks similar internal communication challenges due to within-group differences in education, culture, language and general life experience, but rather are attempting to characterize the ways in which the larger between-group differences may compromise collaboration.

## Common challenges in community–university collaborations

### Language and literacy

Communication challenges can arise when attempting to address specific research questions in researcher–community collaborations. In particular, inconsistencies can exist between scientists and community members in the use of language and definitions. These challenges are most apparent when spoken and written languages differ; for example, Canadian researchers generally communicate in English or French while community members may communicate in a variety of other languages. Communication challenges may still arise, however, even if a common language is used: the choice of vocabulary or phrases can determine how something is communicated and may differ between researchers and community members, particularly when conducting research with a strong biomedical component. These differences can result from diverse education and experience, and they are particularly challenging when scientists and community members are from different cultural and linguistic backgrounds (5–8). In these instances, the gap between ways of talking and thinking about science may be widened, further complicating the interactions and exchanges that are essential for effective collaboration.

Scientists frequently use specialty-specific language. This may include the use of terms or acronyms that are part of a scientist's regular vernacular, or biomedical terms such as “endoscopy”, that may be unfamiliar to researchers in other fields or the general public. Less obviously, speciality-specific language can include familiar words and phrases that are used with a more restricted meaning, for example, the statistical meaning of the word “significant”. Even if speciality-specific language is avoided, scientists may unintentionally speak or write from a certain perspective and with an assumed foundation of knowledge that makes the message inaccessible to community partners. For instance, if researchers are discussing the impact of a bacterium on a population, it may be assumed that there is shared knowledge about what bacteria are. Similarly, confusion may result from differing literacy levels. This may occur through differences in general literacy, subject-specific literacy, or through differences in numerical or statistical literacy which is particularly problematic in public health research where statistics are often used to investigate hypotheses (9).

Community members may also use terms unknown to academic researchers. For example, in an ethnographic study, Cassidy (2008) found that Alaskan Inupiat peoples referred to “bad-blood” as a precursor to, and a product of, cancer (10). The term “bad-blood” may not be widely understood and could be interpreted in a variety of ways. Community members may also refer to geographic landmarks, the names of local people, organizations, or practices that are unfamiliar to researchers. For instance, community members in northern Canada may use the term “living on the land” to describe spending time in handmade cabins outside community limits. To others, this phrase could describe traditional hunting practices, surviving outside of towns or cities, or building shelters from materials available in the natural environment. Similarly, daily life practices in rural communities may not be familiar to researchers. For example, the use of the last 4 digits of 7-digit phone numbers is understood among residents of small communities where the first 3 digits of all phone numbers are the same and thus known to everyone. For researchers, this may not be immediately obvious and may create confusion.

Difficulties may also arise from the inconsistent use of familiar language, where the meaning of commonly used words or phrases may vary. For instance, the term “bug” can be used to describe an insect, a germ, a pest, or an illness such as cold or flu, or even cancer. Every day phrasing may also be disparate. For example, researchers may use the phrase, “What is that?” while community members may state, “What that is?” to ask the same question. Although the meanings may be equivalent, the thought process required to interpret each slightly unfamiliar phrase may make communication disjointed and uncomfortable. The process by which this communication occurs may also be challenging: health scientists may prefer text that provides detailed explanations, and community members may prefer visual diagrams or verbal communication (11).

### Research and expectations

Additional challenges arise from differing perceptions of the investigative process. The term “research” may be defined in a variety of ways, resulting in disparate conceptions about the purpose of research and the process by which it is conducted. Community members may broadly define research as any process of gathering data, information, or facts to form knowledge about a specific topic (12). This may be accomplished through the gathering of information from libraries, news sources, conversations, or other sources, with or without using reproducible protocols for gathering data or applying methods of analysis believed by experts to yield scientifically valid results. Conversely, biomedical or public health scientists typically view research as a rule-governed process that uses systematic observations to test, screen, or form hypotheses. In addition to different perspectives about the definition of research, differences may also exist in perceptions about the purpose of research. Community members may view research as a means to address their concerns and to immediately identify solutions. Alternately, scientists generally view research as a systematic exploration of evidence to address specific research questions.

Divergent definitions and perspectives about research can lead to contrasting expectations about research timelines. Biomedical or public health scientists typically proceed slowly and systematically in putting together pieces of information to support and build knowledge, whereas community members who may be unaware of the time required for many scientific processes may expect research results to be available rapidly. Expectations about research funding requirements may also differ. Biomedical and public health researchers are typically required to adhere to institutional and professional guidelines. These include writing reports and publishing findings in a timely fashion. These demands do not always match the requirements of communities that may wish to evaluate and comment on each data analysis according to their own timelines before results are made public (12).

## Case study: community-driven research on *H. pylori* in northern Canada

**Fig. 1:**
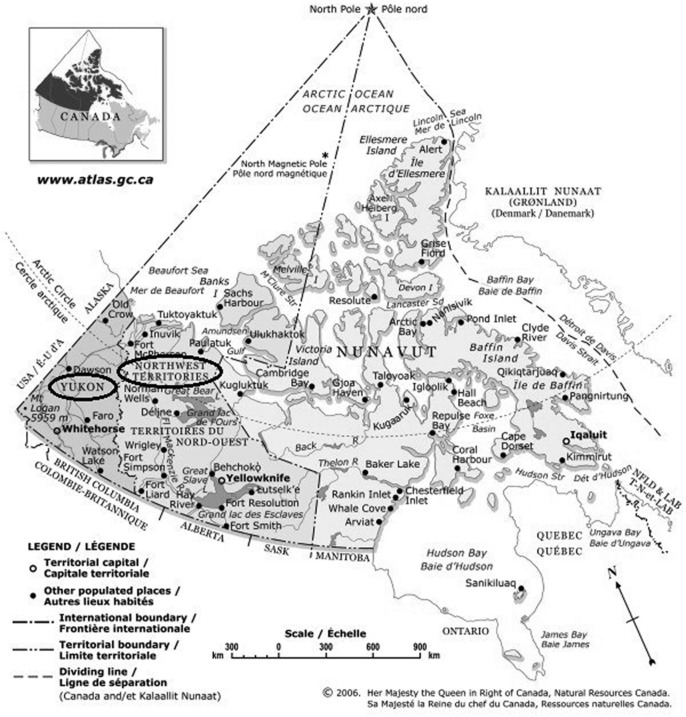
Map of the territories, Canada; Yukon and Northwest territories circled by authors (modified from The Atlas of Canada: the territories, Natural Resources Canada) (10).

*H. pylori* is a bacterium known to persist long-term in the stomach, where it causes chronic gastritis, peptic ulcers and stomach cancer. In northern Aboriginal communities, there is a disproportionately high frequency of *H. pylori* infection and associated diseases, and relatively low success of treatment aimed at eliminating the bacterium (13). Community-driven research is currently underway in northern Aboriginal communities located in the Yukon and Northwest Territories (NWT), Canada (Fig. 1) that aims to describe the burden of disease and risk factors associated with *H. pylori* infection and seeks to identify effective public health strategies for reducing associated health risks (14).

This research links community representatives with faculty from various disciplines such as epidemiology, anthropology, gastroenterology and microbiology at the University of Alberta, as well as territorial health care practitioners and officials. As of 2012, 4 communities were part of this research program: Old Crow, Yukon and Aklavik, Tuktoyaktuk, and Fort McPherson, NWT. Each of these communities is located in a remote area north of the Arctic Circle with populations ranging from approximately 250 to 900, primarily Gwich'in First Nations and Inuvialuit peoples; in the 2006 Canadian Census, over 84% of the residents of each of these communities self-identified as Aboriginal (15). In each case, community representatives invited researchers to work with the community to address concerns related to *H. pylori*. At the beginning of each community research project, and throughout the process, collaborations between researchers and community representatives occur through local planning committees where decisions about research components and implementation are made jointly. This collaborative decision-making process is essential to the success of this research as it ensures that the work done fits with community interests and goals, and that optimal scientific processes are upheld.

Despite our success in these collaborations, communication challenges have arisen. There have been inconsistencies in language used to describe certain terms amongst researchers and community members, including members of the local planning committee, general members of the community and local health care practitioners, as well as news reporters who disseminate information about the research in the local media. For example, regional news reports about our community projects often describe *H. pylori* as a virus. Some community members speak about *H. pylori* as an environmental cancer-causing agent, while others have referred to it as a “worm” in their stomach or as “bad stomach”, raising questions about whether there is a common understanding of the bacterium. Likewise, community members have also referred to the research as work on “HPV” or “H1N1”. It is not clear whether use of these similar acronyms for distinct infectious disease agents are slips of the tongue, whether they reflect differential understandings about the *H. pylori* bacterium, or whether the research itself is not widely understood despite the on-going knowledge exchange activities that are a fundamental component of our community-based, participatory research approach.

There have also been differing views on the purpose of the research. For example, some community members have described the focus of the research on water quality (16) or on pinpointing the source of *H. pylori* in order to eliminate it from the environment. Furthermore, some community members describe the main goal of the research in health care terms as immediate treatment of *H. pylori* infection for all community members found to be infected. Similarly, differences have been apparent in expectations about the research process. Community members have stated that they expected the research to be finished quickly and to receive answers to their questions soon thereafter. This differs from the perspective of biomedical or public health researchers who view research as a process that takes time and yields uncertain results, and thus requires many pieces of a puzzle to come together before generating adequate evidence on which to base solutions to complex problems.

In these community-based *H. pylori* research projects, researchers have worked to alleviate these issues through on-going engagement with community partners via local planning committees, and by developing project-specific research agreements to document a shared understanding of the research process and expectations. Researchers and community partners have also worked to promote bi-directional communication that supports the production of meaningful results, aiming to effectively move the knowledge created through research into implementation by users such as community members and healthcare providers—a process known as knowledge translation. In this setting, effective communication between scientists and non-scientists has been a major priority of the collaborative research endeavour because different understandings of language, terminology and expectations of the research process and goals have the potential to negatively impact the relationships that have been built between partners involved in this community-based, participatory health research. In the next section, we will discuss potential consequences to not resolving communication challenges, and present solutions in progress.

## Common consequences of communication challenges in community–university collaborations

Communication challenges resulting from differences in language and differing perspectives about research may lead to misunderstandings between researchers and community members that can jeopardize the research process and damage essential partnerships. At the very least, communication challenges slow the research process: addressing misunderstandings as they arise takes time away from the main research goals. Similarly, it takes time to carefully decipher the meanings of words and expressions used by others so that miscommunication may be avoided so that the research can move forward with all partners on common ground (6,17).

Furthermore, without a shared understanding of language and process, the success of a joint research endeavour may be in jeopardy. Researchers may misunderstand or misinterpret the issues raised by community members and may inadvertently work to answer research questions that are not those posed by the community. Even with agreement about the main purpose of the research, the implementation of this work may not be mutually understood. If so, the work conducted may not incorporate the community context, or may be culturally insensitive (7,8,18). Consequently, research results may not be meaningful to or accepted by community members. If the knowledge gained through the research process is not employed by end-users such as community members, the opportunity for a successful collaboration is lost, as is the time and the resources required to carry out these research endeavours.

Differing definitions and expectations of the research process may also strain relationships and create conflict. Misunderstandings about how the results will ultimately impact the community can occur. For example, community members may believe that for research to be considered successful, it should have recognizable benefits available quickly to the community, whereas scientists may view small gains that contribute to larger bodies of evidence as successes, even if they do not provide solutions immediately. Likewise, divergent definitions or expectations about the partnership itself may create conflict. For instance, a common view among Canadian First Nations communities is that the community has collective ownership of any data collected as part of research collaborations (12). Similarly, partners may disagree on the appropriate custody and allotment of research funds. Conflicts may then occur between researchers bound by institutional and professional expectations if they do not coincide with the expectations of collaborating communities (7).

## Solutions to common communication challenges in community–university collaborations

Research collaborations that include community partnerships can offer a variety of benefits, but challenges can occur when partners are not communicating effectively. Communication challenges may be overcome through the development of knowledge translation and communication strategies and tools, acknowledgement of the importance of trust and reciprocity in these relationships, and through the development of project-specific research agreements that are drafted jointly by researchers and their community partners.

The development of effective knowledge translation and communication tools for research involving scientist–community partnerships will support collaborations throughout the entire research process (19,20). Enhancing effective communication will foster a mutual understanding of one another's perspectives and ultimately help to build strong relationships that are vital to the success of these research endeavours. This may involve an evaluation of whether a common language is used when discussing research components (17), development of a common language between researchers and community members (21), or an assessment of the levels of literacy amongst collaborators and end-users (22). Engagement of local professionals working in the community, such as health care providers and teachers, who can help facilitate understanding between outsider scientists and local residents, is essential to this effort. The identification and development of effective communication tools can also foster a mutual understanding about the research process, methods, results and interpretation amongst all research partners and participants. For example, an evaluation of which communication media, such as community presentations or newsletters, would best facilitate research dissemination could help to enhance successful communication (17,18). Identifying best methods for the dissemination of research results that include statistics may be particularly important: evidence suggests that statistical literacy is low amongst members of the general public (9,23). Presenting statistical results in a way that increases accessibility and transparency could help prevent miscommunication and promote mutual understanding (9). Effective knowledge translation and communication tools will help collaborators identify which results are most meaningful, as well as the most effective ways in which they may be communicated to end-users.

Another way that these research collaborations can be supported is through recognition of the importance of trust and reciprocity to relationship-building (17,24). Trust and reciprocity between researchers and community members will promote meaningful engagement and increase the likelihood that the research will be considered successful (7,24). Funding agencies that support these collaborations should recognize the need for resources required for the development of strong community–researcher relationships (6,17). The need for financial support for costs of relationship-building is particularly crucial for collaborative research endeavours involving long-distance partnerships where communication is often accomplished over email or by telephone. Successful relationship-building takes time, and the development and maintenance of trust in community-based work is typically more successful and fulfilling when done in person (17,19,24). As such, researchers conducting work involving community collaborations should allow for multiple in-person discussions when planning and budgeting. Researchers and funders should also consider that the respect and reciprocity that are necessary for strong and meaningful relationships require a mutual exchange of privileges; a balanced distribution of resources and power is imperative (7,8,12,25).

Communication challenges may also be overcome through the collaborative development of research agreements. The process of developing a research agreement requires collaborative partners to begin developing shared language and expectations before the research is started (26). Research partners should discuss the overall goals of the collaboration and include a statement of objectives at the beginning of the agreement; this provides context for interpreting the agreement (27). The parties should also discuss their expectations of the research (including benefits, contributions and timelines), and ensure that provisions of the agreement are consistent with a shared-understanding of expectations. The agreement should include a list of definitions used in the document (28). This process will help open communication about a variety of issues that may not have otherwise been discussed, and help prevent communication challenges later on by providing written documentation that partners can consult over time.

## Important consideration

The challenges outlined and solutions suggested in this article come from the perspective of researchers involved in community-driven health research with a substantial biomedical component. It is expected that researchers from different disciplines would have a different perspective and consider different solutions. Likewise, community partners participating in research initiatives, such as those involved in the case study outlined here, likely have different views on the challenges that exist in addressing their concerns, and on the potential ways to improve communication and research partnerships.

## Conclusions

Communication challenges may arise when community members and researchers work together to answer community health research questions using biomedical or public health methods of inquiry. From a health researcher's perspective, these challenges include inconsistencies in the use of language, as well as differences in the values and expectations about the research process. These result, in part, from differences in ways of thinking about science and are deepened by differences in culture and language. If research partners do not address different understandings of language and the research process, partners may risk damaging their relationships and slowing down progress on research questions, which have the potential to impact real-world problems. When conducting public health research with a strong biomedical component, effective engagement and relationship-building, reinforced by formal research agreements between collaborators, as well as development of knowledge translation and communication tools may all work to increase shared understanding and ultimate success in collaborations between scientists and community members.
